# A CREB1/miR-433 reciprocal feedback loop modulates proliferation and metastasis in colorectal cancer

**DOI:** 10.18632/aging.101671

**Published:** 2018-12-06

**Authors:** Li Yan, Wei-Qiang You, Neng-Quan Sheng, Jian-Feng Gong, Lan-Dian Hu, Ge-Wen Tan, Hong-Qi Chen, Zhi-Gang Wang

**Affiliations:** 1 Department of General Surgery, Shanghai Jiao Tong University Affiliated Sixth People’s Hospital, Shanghai, China; 2 Shanghai Institute of Nutrition and Health, Chinese Academy of Sciences, Shanghai, China; * Equal contribution

**Keywords:** miR-433, CREB1, CCAR1, JNK1, colorectal cancer

## Abstract

Increasing evidence has indicated the prognostic value of miR-433 across a series of malignancy types. However, the underlying mechanisms involved in cancer progression haven’t been sufficiently elucidated. In the present work, we found that miR-433 was downregulated in CRC tissues and cell lines. Ectopic expression of miR-433 obviously suppressed the proliferation, invasion and metastasis activity of CRC cells in vitro and in vivo. CREB1, CCAR1 and JNK1 were highly expressed and negatively correlated with miR-433 expression in CRC. CRC patients with higher expression of CREB1, CCAR1 or JNK1 presented a worse outcome relative to those with lower expression. CREB1 transactivated the expression of miR-433, and CREB1, CCAR1 and JNK1 simultaneously served as its targets, which in turn composed a feedback loop between CREB1 and miR-433. miR-433 blocked cell cycle progression and abolished EMT. Collectively, our study demonstrated the CREB1/miR-433 reciprocal feedback loop restrained the propagation, invasion and metastasis activities of CRC cells through abrogation of cell cycle progression and constraint of EMT.

## Introduction

Colorectal cancer (CRC) is one of the most common malignancies worldwide in morbidity and mortality [1–3]. With the comprehensive development of surgery aligned with chemoradiotherapy, molecular targeted agents and immunotherapy, the five-year overall survival (OS) of CRC patients has achieved a superior outcome in the past few decades. However, almost one half of patients will inevitably progress to liver metastasis disease, synchronously or metachronously [4,5]. Upon metastasis, the five-year OS rate does not exceed 10% [6], which is so unsatisfactory for patients and physicians. Even though, unfortunately, most of stage IV patients who successfully receive radical hepatectomy will unavoidably relapse due to chemoresistance. Given the treatment dilemma, it’s urgent to illuminate the mechanisms involved in chemoresistance and to discover some novel predictive biomarkers and molecular reagents.

Typically, cAMP regulatory element-binding protein (CREB1) is overexpressed in a series of human neoplasms, and it can be activated through phosphorylation by a number of kinases, such as Akt (serine/threonine kinase 1), p90Rsk (ribosomal protein S6 kinase A1, RPS6KA1), protein kinase A (PKA), and calcium/calmodulin-dependent kinases. Activated CREB1 regulates proliferation and cell survival by binding to the promoter of target genes dependent on disparate stimuli. Cyclins, Bcl-2 family members, and Egr-1(early growth response 1) are viewed as canonical target genes of CREB1 [7]. Meanwhile, the cAMP/CREB1 pathway is involved in 5-FU (5-fluoro-2,4(1H, 3H)-pyrimidinedione) and platinum-resistance in many types of solid tumors [8–10]. Therefore, CREB1 could be a promising molecular target in the future. Recently, some researches have indicated that CREB1 could regulate cancer progression through transcriptional modulation of microRNA. Pan and colleagues reported that CREB1 transactivated miR-196-5p by binding to its promotor in bladder cancer [11].

MicroRNA433 (miR-433) is located on chr14q32.31. Recent studies have revealed its predictive value in prognosis. Zheng et al. demonstrated that miR-433 was downregulated and correlated with poor prognosis in gastrointestinal cancers [12]. Similarly, Ueda and colleagues showed that low expression of miR-433 in gastric cancer was associated with an unfavorable overall survival outcome independent of clinical covariates, including depth of invasion, lymph node metastasis, and stage [13]. Analogous results were reported in hepatocellular carcinoma, prostate cancer, pleural malignant mesothelioma and glioblastoma [14–18]. Although the prognostic value of miR-433 has been well mined, the mechanism underlying its involvement in CRC progression has not been sufficiently elucidated.

In this present work, we explored the targets of miR-433 and its upstream regulon in CRC tissues and cell lines in vitro and in vivo. We found that miR-433 suppressed proliferation, invasion, subcutaneous tumorigenesis and metastasis. CREB1, CCAR1 and JNK1 were direct targets of miR-433, and the transcription factor CREB1 transactivated miR-433 expression by binding to its promoter region. In other words, CREB1/miR-433 composed a reciprocal feedback loop to regulate the progression of CRC.

## RESULTS

### Downregulation of miR-433 participates in the progression of CRC

**Figure 1:**
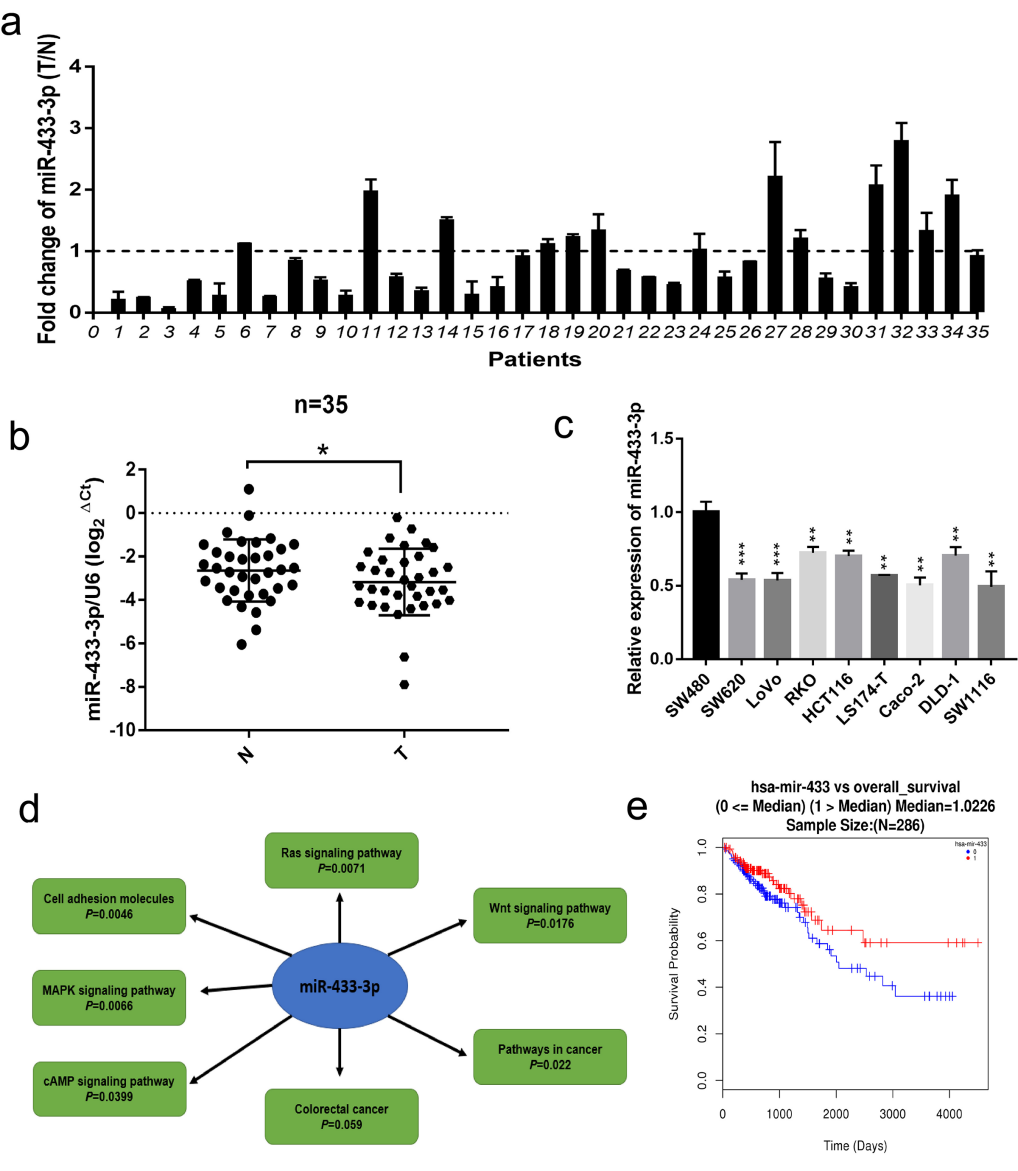
**miR-433 was downregulated in CRC specimens and cell lines and correlated with signaling pathway associated with CRC progression.** (**a**) The relative expression ratio of miR-433 in 35 paired fresh CRC and adjacent normal mucosa specimens (T, tumor; N, adjacent normal mucosa). (**b**) miR-433 was significantly underexpressed in CRC than adjacent normal mucosa by qRT-PCR (miR-433 referenced to U6). (**c**) The expression of miR-433 in several CRC cell lines. (**d**) The signaling pathways connected with miR-433 were mined in the miRWalk 3.0 database. (**e**) A survival curve relative to miR-433 expression median in CRC was plotted using the LinkedOmics database. *, *p*<0.05; **, *p*<0.01; ***, *p*<0.001.

Firstly, we conducted qRT-PCR in 35 paired fresh colorectal cancer and adjacent normal mucosa specimens. As displayed in Figure 1a-b, miR-433-3p exhibited lower expression in cancer relative to normal mucosa. We then performed real-time PCR in CRC cell lines, as shown in Figure 1c, and found that miR-433-3p tended to present relatively low expression across the 9 cell lines. The miRWalk database was adopted to carry out the KEGG (Kyoto Encyclopedia of Genes and Genomes) pathway analysis, and the results indicated that miR-433 is significantly correlative to the Wnt, Ras, MAPK, cAMP signaling pathways and cell adhesion molecules, which are commonly mis-regulated in cancer progression (Figure 1d). Last, we utilized the LinkedOmics database to determine the prognostic value of miR-433 in CRC and found that higher expression of miR-433 tended to be a protective variable in prognosis, but not significantly (Figure 1e).

### miR-433 suppresses CRC cell proliferation in vitro and in vivo

**Figure 2:**
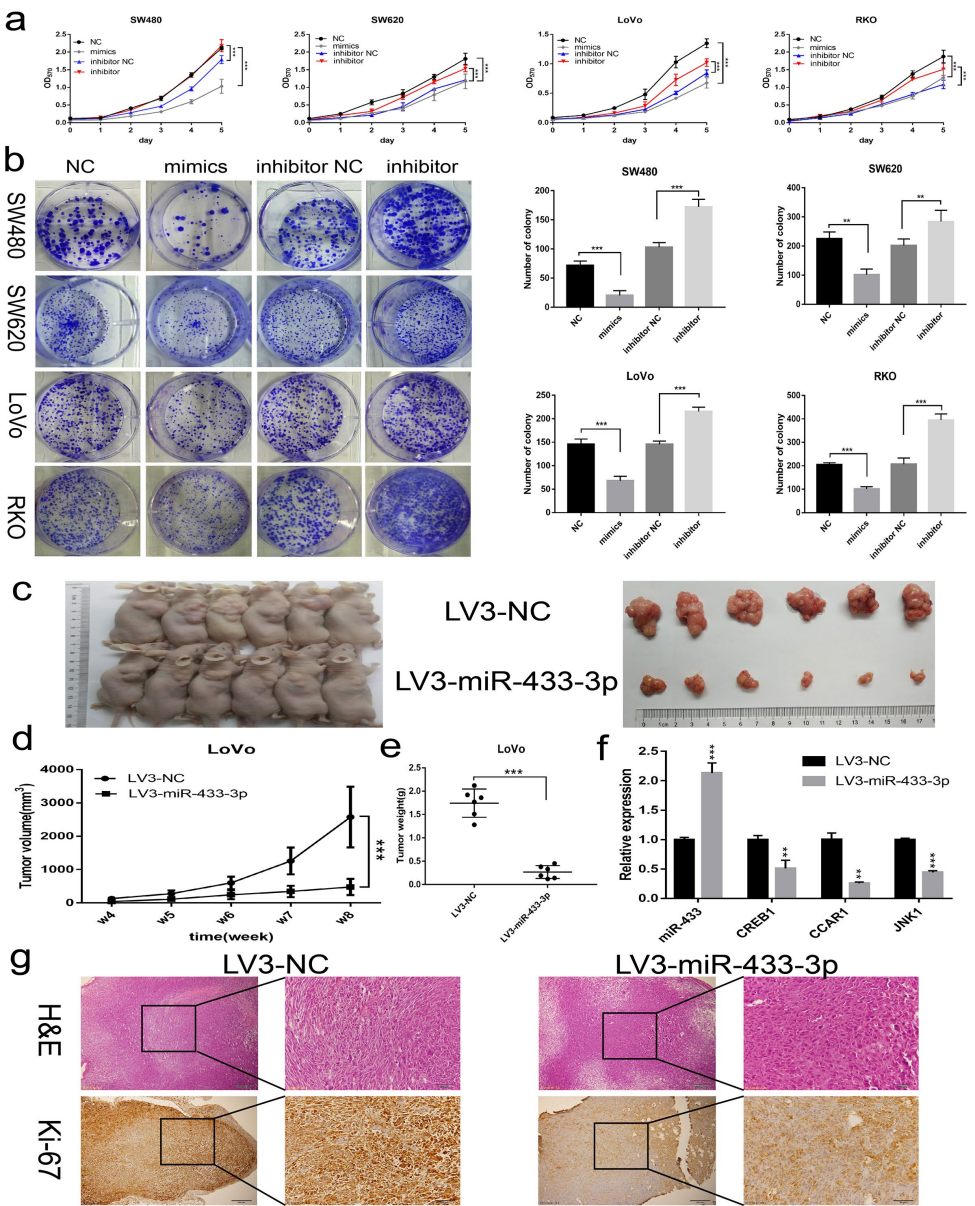
**miR-433 inhibited CRC cell proliferation in vitro and in vivo.** (**a**) MTT assays revealed that transfection of miR-433 mimics evidently suppressed the cell viability in SW480, SW620, LoVo and RKO cells, however, miR-433 inhibitor conspicuously enhanced the proliferative activity relative to inhibitor NC (NC, negative control; mimics, miR-433 mimics; inhibitor NC, negative control for inhibitor; inhibitor, inhibitor of miR-433). (**b**) Colony formation assays indicated that overexpression or knockdown of miR-433 prominently impeded or promoted CRC cell colony formation activity via transfection of the mimics or inhibitor of miR-433 (NC, negative control; mimics, miR-433 mimics; inhibitor NC, negative control for inhibitor; inhibitor, inhibitor of miR-433). (**c**) Subcutaneous tumors generated in nude mice which derived from LV3-NC- and LV3-miR-433-3p-infected LoVo cells are shown. (**d**) ANOVA of repeated measurements confirmed that the LoVo/LV3-miR-433-3p group showed much feebler growth than its counterpart. (**e**) A *t* test demonstrated a significant difference in tumor weight between the LoVo/LV3-miR-433-3p group and its counterpart. (**f**) Real-time PCR indicated that miR-433 was upregulated in LoVo/LV3-miR-433-3p tumors, and subsequently, a dramatic decline in CREB1, CCAR1 and JNK1 was observed. (**g**) H&E and Ki-67 staining of tumors initiated from LoVo/LV3-NC and LoVo/LV3-miR-433-3p cells. **, *p*<0.01; ***, *p*<0.001.

To assess the biological impact of miR-433 on CRC cells, gain-of-function and knockdown approaches were carried out. As shown in Figure 2a, miR-433-3p mimics notably repressed SW480, SW620, LoVo and RKO cells propagation compared with NC, and its inhibitor conferred more robust cell viability on these cells relative to the counterpart groups. Similarly, miR-433-3p mimics and inhibitor remarkably attenuated or reinforced the colony formation ability of CRC cells compared with their respective control groups (Figure 2b). Then, we cloned a miR-433-3p overexpression vector into lentivirus and infected LoVo cells to carry out subcutaneous oncogenesis. From the fourth week, we repeatedly measured the diameter of the subcutaneous tumors in the nude mice. With time elapsing, the tumors in the LoVo/LV3-NC group grown more and more robustly compared with the counterpart. Eight weeks after inoculation, the mice were sacrificed. As presented in Figure 2c, nude mice in the LoVo/LV3-miR-433-3p group apparently formed smaller tumors than those in the counterpart group. The tumor growth curve and weights between the LV3-miR-433-3p and LV3-NC groups were also significantly different (Figure 2d-e). The mean tumor volume of LV3-NC group was more than quintuple than that in the counterpart group (2575.76 ± 909.63 vs 476.81 ± 245.58 mm^3^). Furthermore, the mean tumor weight of LV3-NC group was more than sextuple than that in the LoVo/LV3-miR-433-3p group (1.74 ± 0.30 vs 0.27 ± 0.14 g). Hematoxylin and eosin stained slides were imaged and are displayed in Figure 2g. IHC revealed less Ki-67 staining in LoVo/LV3-miR-433-3p tumors relative to counterparts.

### miR-433 abrogates CRC invasion and liver metastasis in vitro and in vivo

**Figure 3:**
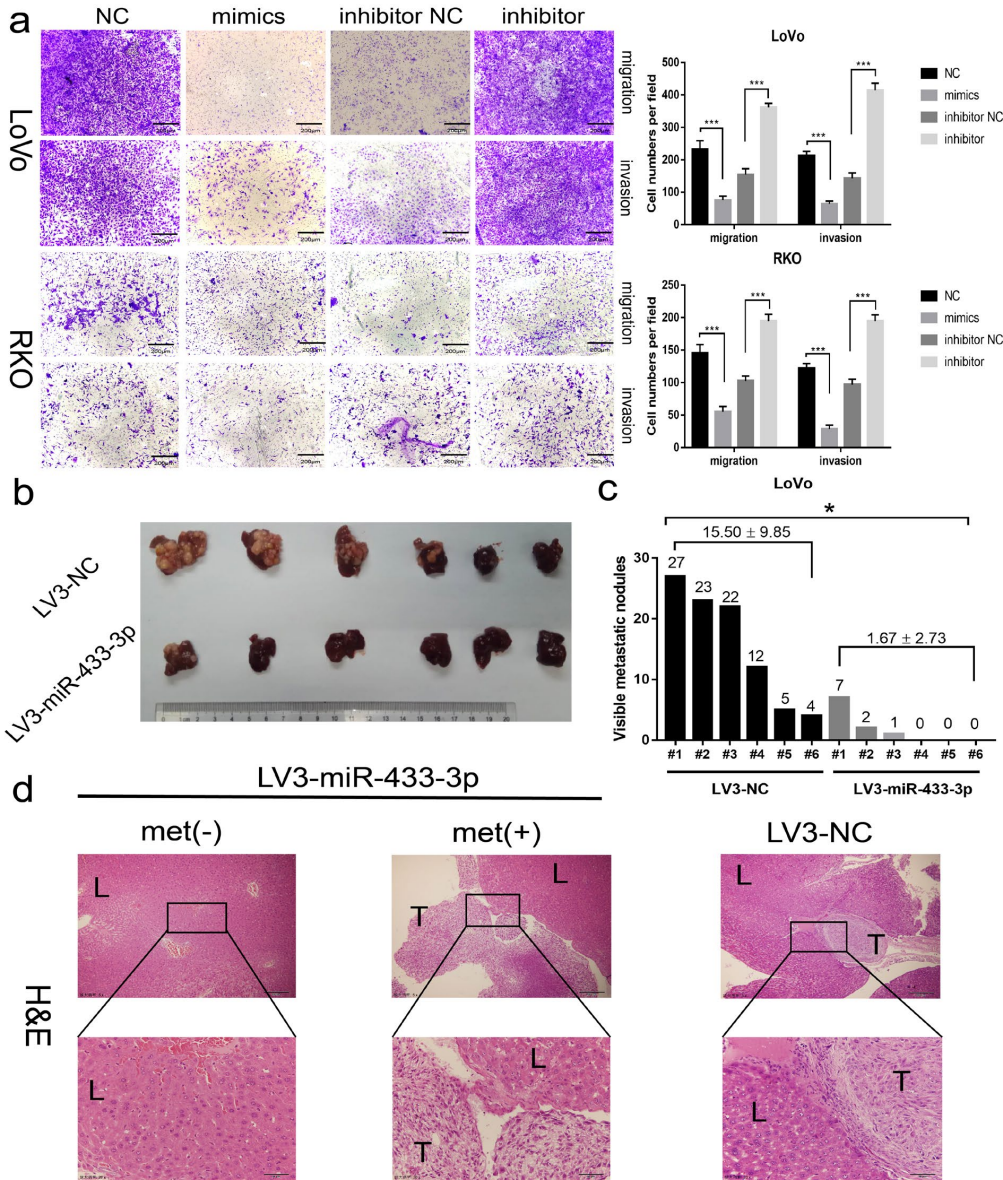
**miR-433 attenuated the invasion and metastasis properties in CRC.** (**a**) Transwell assays showed that restoration or downregulation of miR-433 by transfection of the mimics or inhibitor of miR-433 remarkably rescinded or reinforced the migration and invasion phenotype in LoVo and RKO cells (NC, negative control; mimics, miR-433 mimics; inhibitor NC, negative control for inhibitor; inhibitor, inhibitor of miR-433). (**b**) Four weeks after intra-splenic injection of LoVo/LV3-miR-433-3p and LoVo/LV3-NC cells, the livers of the two group mice were collected and photographed. (**c**) Corresponding visible metastatic nodules in the two groups showed that LV3-miR-433-3p conspicuously abolished the metastasis activity of LoVo cells. (**d**) H&E-stained livers with or without metastases were imaged (met(-), negatively metastatic mouse livers; met(+), positively metastatic mouse livers; T, metastatic tumor; L, liver). *, *p*<0.05; ***, *p*<0.001.

As shown in Figure 3a and S3, miR-433-3p mimics obviously inhibited the migration and invasion activity of LoVo and RKO cells compared with NC; and miR-433-3p inhibitor upregulated the cell migration and invasion properties relative to inhibitor NC groups. An intra-spleen injection CRC liver metastasis model was performed in mice, as exhibited in Figure 3b-c, and the LoVo/LV3-miR-433-3p group presented much fewer visible metastatic nodules than the LoVo/LV3-NC group on the mouse livers. H&E staining demonstrated that the tissues that infiltrated into the liver was derived from LoVo cells (Figure 3d).

### CREB1, CCAR1 and JNK1 are direct targets of miR-433 in CRC

**Figure 4:**
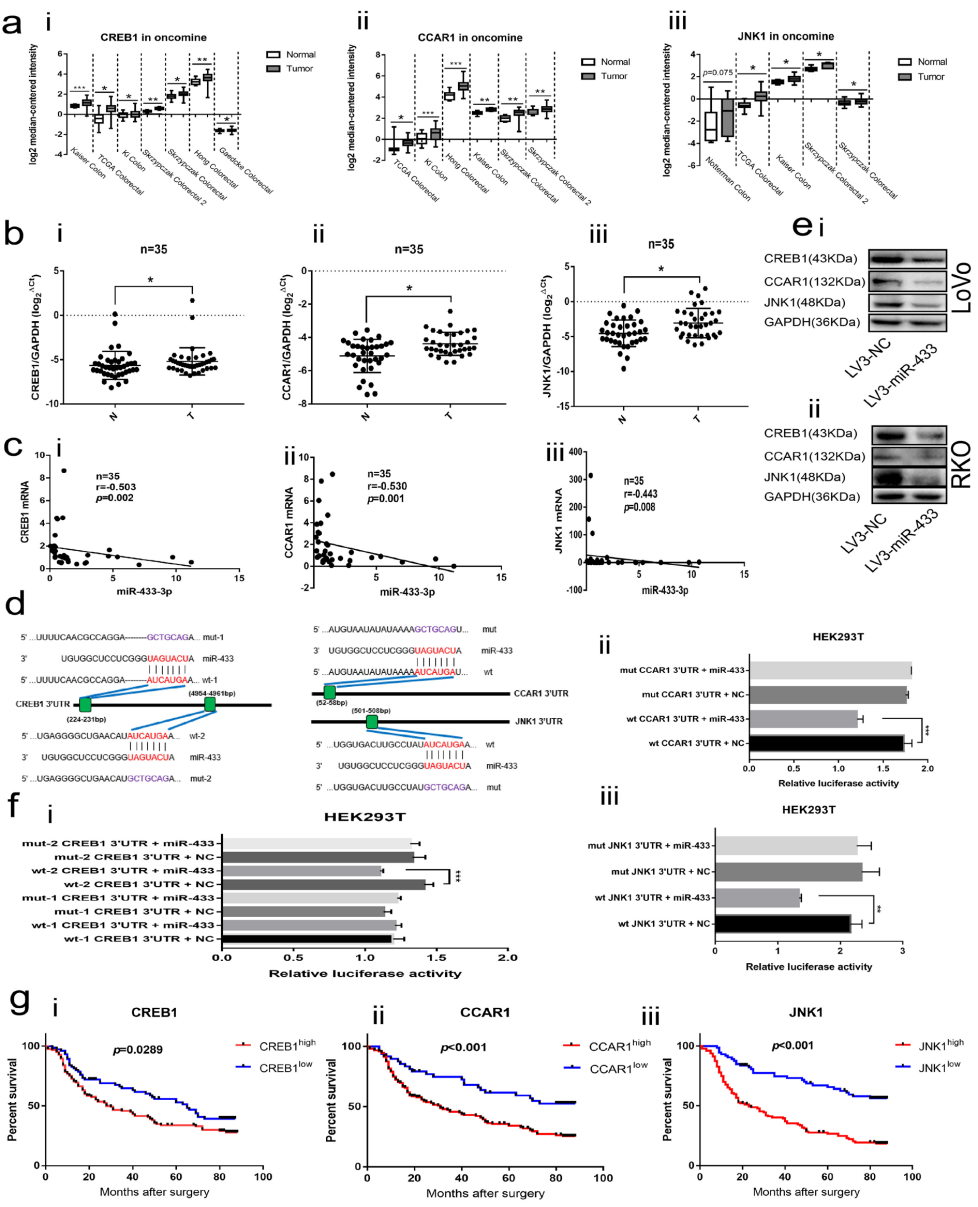
**CREB1, CCAR1 and JNK1 were the direct targets of miR-433.** (**a**) Bioinformatics analyses showed overexpression of CREB1 (i), CCAR1 (ii) and JNK1 (iii) in the Oncomine database. (**b**) Real-time PCR manifested upregulation of CREB1 (i), CCAR1 (ii) and JNK1 (iii) in 35 paired CRC and normal mucosa specimens. (**c**) Bi-variable correlation analyses revealed that CREB1 (i), CCAR1 (ii) and JNK1 (iii) were all significantly correlated with miR-433 in 35 paired tissues. (**d**) A schematic of the putative binding sites between miR-433 and its targets according to the TargetScan database. (**e**) Western blot illustrating that miR-433 inhibited the expression of CREB1, CCAR1 and JNK1 in LoVo (i) and RKO (ii) cells. (**f**) Dual luciferase assays showing that miR-433 directly targets CREB1 (i), CCAR1 (ii) and JNK1 (iii). (**g**) Kaplan-Meier analysis combined with the log-rank method indicated that CRC patients with higher expression of CREB1 (i), CCAR1(ii) or JNK1 (iii) had a poorer prognosis. *, *p*<0.05; **, *p*<0.01; ***, *p*<0.001.

The Oncomine database was applied to explore the expression of CREB1, CCAR1 and JNK1 in CRC tissues. As illustrated in Figure 4a, CREB1, CCAR1 and JNK1 were all overexpressed in CRC relative to normal tissues across a series of subsets. Next, we conducted real-time PCR in our collected 35 paired fresh CRC and adjacent normal mucosa specimens, and a consistent result was observed (Figure 4b). Meantime, Spearman correlation analyses revealed that miR-433-3p is negatively correlated with CREB1, CCAR1 and JNK1 mRNA levels (Figure 4c).

The TargetScan database was adopted to predict the binding sites between miR-433 and its targets. As shown in Figure 4d, two putative binding sites existed in the 3’UTR region of CREB1; however, only a single conserved site presented in the 3’UTR of CCAR1 and JNK1. Likewise, a negatively correlated model between CREB1, CCAR1, JNK1 and miR-433 was observed when we up- or down-regulated miR-433 through transient or stable transfection in CRC cells at the mRNA and protein level (Supplementary Figure S1a-b and Figure 4e). Furthermore, the same phenomenon was again found in the subcutaneous tumors (Figure 2f).

The dual luciferase assays were used to determine the targeted binding activity between miR-433 and its putative targets. As shown in Figure 4f, there was no difference between the wt-1 CREB1 3’UTR and mut-1 CREB1 3’UTR. Inversely, a remarkable decrease in luciferase activity was found when wt-2 CREB1 3’UTR was co-transfected with the miR-433 mimics, which indicated that miR-433 targeted CREB1 by binding to the second presumed site. Positive binding results were also observed among wt CCAR1 3’UTR, wt JNK1 3’UTR and miR-433 mimics.

Next, we conducted IHC in 200 FFPE CRC samples. An immune-staining intensity score system was adopted as mentioned above. Representative images of the hierarchic staining of CREB1, CCAR1 and JNK1 are displayed in Supplementary Figure S2. As shown in Table 1, chi-square analyses revealed no evident association between CREB1 and clinicopathological factors, although CREB1 appeared to be connected with distant metastasis. CCAR1 was relative to tumor depth, distant metastasis and pTNM stage, but not to age, gender, tumor location, tumor size, histological grade, lymph node invasion, CEA, CA125 or CA19-9. JNK1 was correlated with lymph node invasion, pTNM stage and CA19-9, but not with age, gender, tumor location, tumor size, histological grade, tumor depth, distant metastasis, CEA or CA125.

**Table 1:** Correlation between clinicopathological parameters and CREB1/CCAR1/JNK1 expression levels in 200 CRC patients

**Variables**	**CREB1**		***p***	**CCAR1**		***p***	**JNK1**		***p***
	Low(%)	High(%)		Low(%)	High(%)		Low(%)	High(%)	
Age <60 ≥60	23(30.7) 52(69.3)	39(31.2) 86(68.8)	0.937	15(31.3) 33(68.8)	47(30.9) 105(69.1)	0.966	22(30.1) 51(69.9)	40(31.5) 87(68.5)	0.841
Gender Male Female	42(56) 33(44)	72(57.6) 53(42.4)	0.825	30(62.5) 18(37.5)	84(55.3) 68(44.7)	0.377	42(57.5) 31(42.5)	72(56.7) 55(43.3)	0.908
Tumor location Colon Rectum	52(69.3) 23(30.7)	90(72.0) 35(28.0)	0.687	30(62.5) 18(37.5)	112(73.7) 40(26.3)	0.137	52(71.2) 21(28.8)	90(70.9) 37(29.1)	0.956
Tumor size <15cm^3^ ≥15cm^3^	26(34.7) 49(65.3)	38(30.4) 87(69.6)	0.531	19(39.6) 29(60.4)	45(29.6) 107(70.4)	0.196	27(37.0) 46(63.0)	37(29.1) 90(70.9)	0.252
Differentiation Well& moderate Poor	54(72.0) 21(28.0)	101(80.8) 24(19.2)	0.149	38(79.2) 10(20.8)	117(77.0) 35(23.0)	0.751	56(76.7) 17(23.3)	99(78.0) 28(22.0)	0.840
Tumor depth T1-T2 T3-T4	8(10.7) 67(89.3)	18(14.4) 107(85.6)	0.447	11(22.9) 37(77.1)	15(9.9) 137(90.1)	**0.019**	13(17.8) 60(82.2)	13(10.2) 114(89.8)	0.125
Lymph node invasion Absent Present	37(49.3) 38(50.7)	63(50.4) 62(49.6)	0.884	28(58.3) 20(41.7)	72(47.4) 80(52.6)	0.185	49(67.1) 24(32.9)	51(40.2) 76(59.8)	**<0.001**
Distant metastasis Absent Present	66(88.0) 9(12.0)	98(78.4) 27(21.6)	0.087	44(91.7) 4(8.3)	120(78.9) 32(21.1)	**0.046**	63(86.3) 10(13.7)	101(79.5) 26(20.5)	0.230
pTNM I-II III-IV	32(42.7) 43(57.3)	56(44.8) 69(55.2)	0.769	27(56.3) 21(43.8)	61(40.1) 91(59.9)	**0.0499**	45(61.6) 28(38.4)	43(33.9) 84(66.1)	**<0.001**
CEA <5µg/l ≥5µg/l	42(56.0) 33(44.0)	75(60.0) 50(40.0)	0.578	32(66.7) 16(33.3)	85(55.9) 67(44.1)	0.188	47(64.4) 26(35.6)	70(55.1) 57(44.9)	0.200
CA125 <35U/ml ≥35U/ml	56(74.7) 19(25.3)	86(68.8) 39(31.2)	0.376	35(72.9) 13(27.1)	107(70.4) 45(29.6)	0.737	56(76.7) 17(23.3)	86(67.7) 41(32.3)	0.177
CA19-9 <37U/ml ≥37U/ml	51(68.0) 24(32.0)	87(69.6) 38(30.4)	0.813	37(77.1) 11(22.9)	101(66.4) 51(33.6)	0.165	59(80.8) 14(19.2)	79(62.2) 48(37.8)	**0.006**

*p*< 0.05, chi-square test. pTNM: pathological tumor lymph node metastasis classification; CEA: carcinoembryonic antigen; CA125: carbohydrate antigen-125; CA19-9: carbohydrate antigen-19-9.

Then, a Cox proportional hazard regression model was established. As shown in Table 2, univariate analysis revealed that tumor depth, lymph node invasion, distant metastasis, pTNM stage, CEA, CA19-9, CREB1, CCAR1 and JNK1 may act as potential prognosticators. In light of that the pTNM stage system integrates the clinical significance of the tumor, lymph node and distant metastasis, only pTNM stage, CEA, CA19-9, CREB1, CCAR1 and JNK1 were incorporated into the multivariate analyses. The results indicated that pTNM stage, CEA, CCAR1 and JNK1 could serve as independent variables in prognosis. In addition, CREB1 was another potent borderline candidate.

**Table 2:** Univariate and multivariate analyses of the variables associated with overall survival of patients with CRC

**Variables**	**Univariate**		**Multivariate**	
	**HR(95%CI)**	***p***	**HR(95%CI)**	***p***
Age (≥60 vs < 60)	1.256(0.858-1.839)	0.241	-	-
Gender (Male vs Female)	1.199(0.844-1.704)	0.310	**-**	**-**
Tumor location (Colon vs Rectum)	1.169(0.796-1.717)	0.427	**-**	**-**
Tumor size (≥15 vs <15cm^3^)	1.429(0.970-2.106)	0.071	**-**	**-**
Differentiation (Poor vs Well& moderate)	1.021(0.676-1.544)	0.920	**-**	**-**
Tumor depth (T3-T4 vs T1-T2)	4.069(1.896-8.731)	**<0.001**	*NA*	*NA*
Lymph node invasion (Present vs Absent)	3.097(2.148-4.465)	**<0.001**	*NA*	*NA*
Distant metastasis (Present vs Absent)	4.305(2.807-6.602)	**<0.001**	*NA*	*NA*
pTNM (III-IV vs I-II)	3.708(2.516-5.465)	**<0.001**	2.851(1.892-4.297)	**<0.001**
CEA (≥5 vs <5µg/l)	2.048(1.450-2.893)	**<0.001**	1.678(1.157-2.431)	**0.006**
CA125 (≥35 vs <35U/ml)	1.325(0.915-1.918)	0.137	**-**	**-**
CA19-9 (≥37 vs <37U/ml)	1.511(1.050-2.173)	**0.026**	1.023(0.692-1.513)	0.908
CREB1 (high vs low)	1.493(1.036-2.153)	**0.032**	1.458(0.999-2.128)	0.051
CCAR1 (high vs low)	2.128(1.343-3.372)	**0.001**	1.699(1.058-2.728)	**0.028**
JNK1 (high vs low)	3.054(2.024-4.607)	**<0.001**	2.062(1.330-3.197)	**0.001**

Cox proportional regression model was performed. *NA,* not adopted. *p* < 0.05.

Last, Kaplan-Meier analyses combined with the log-rank method demonstrated that the patients with higher expression of CREB1, CCAR1 or JNK1 were predisposed to a much shorter OS than those with lower expression (Figure 4g).

### CREB1 transactivates miR-433 by binding to its promoter

**Figure 5:**
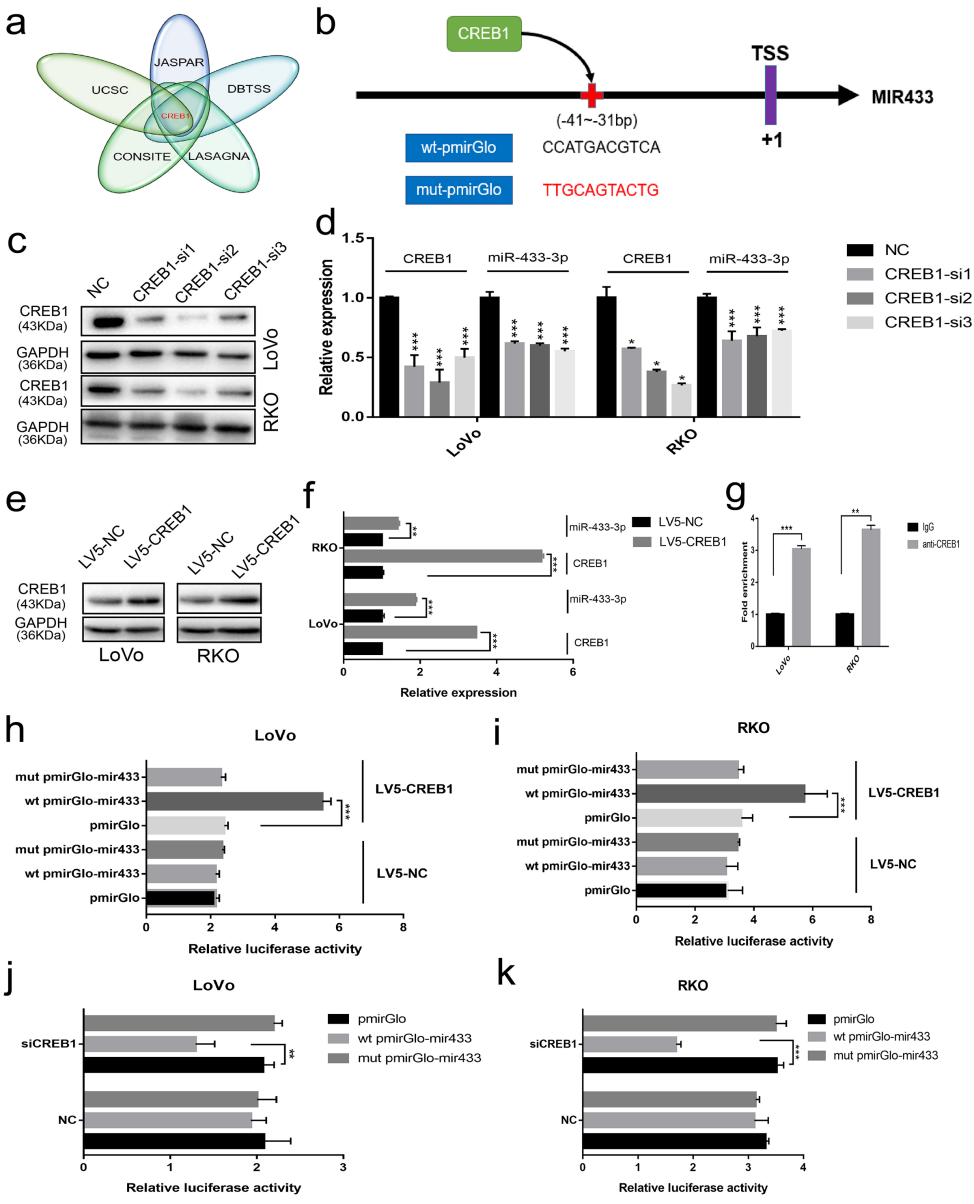
**CREB1 transactivated the expression of miR-433.** (**a**) UCSC aligned DBTSS, JASPAR, CONSITE and LASAGNA databases demonstrated that CREB1 could bind to the promotor region of miR-433. (**b**) The predicted binding sites and mutation sequences are presented. (**c-d**) Downregulation of miR-433-3p was observed when CREB1 was silenced with siRNA at the mRNA and protein level in LoVo and RKO cells through real-time PCR and western blot. (**e-f**) The expression of miR-433-3p was subsequently upregulated after overexpression of CREB1 in LoVo and RKO cells by qRT-PCR and western blot. (**g**) ChIP-qPCR assay indicated that anti-CREB1 enriched much more DNA fragment which contains putative CREB1 binding site on the miR-433 promotor relative to IgG. (**h-k**) Luciferase assays confirmed the specific targeting relationship between CREB1 and the miR-433 promotor (pmirGlo, empty luciferase reporter vector; wt pmirGlo-mir433, wild-type luciferase reporter plasmid of miR-433 promotor containing putative CREB1 binding site; mut pmirGlo-mir433, luciferase reporter plasmid of miR-433 promotor which mutated putative CREB1 binding site; LV5-NC, lentivirus package of empty vector; LV5-CREB1, lentivirus package of CREB1 overexpressing plasmid). *, *p*<0.05; **, *p*<0.01; ***, *p*<0.001.

To elucidate the underlying mechanism involved in the downregulation of miR-433 in CRC, we considered the possibility of transcriptional modulation. The DBTSS database was utilized to explore the presumed promotor region of miR-433. The corresponding sequence was acquired from the UCSC gene browser. Finally, a combination approach using JASPAR, CONSITE and LAGASNA databases was adopted to predict the putative transcription factor binding site. As demonstrated in Figure 5a, all three of the datasets predicted a probable CREB1 binding site. Therefore, we purchased wt-pmirGlo-mir433 and mut-pmirGlo-mir433 luciferase vectors from GenePharma (Figure 5b).

Next, we found that the expression of miR-433-3p was downregulated when we successfully knocked down CREB1 at the mRNA and protein level (Figure 5c-d). A similar result was also discovered when we detected the expression of miR-433 after LV5-CREB1 infection of LoVo and RKO cells (Figure 5e-f). As shown in Figure 5g, a ChIP-qPCR assay confirmed that anti-CREB1 could enrich much more DNA fragment containing putative CREB1 binding site on the miR-433 promotor region relative to IgG. Luciferase assays indicated that overexpression of CREB1 could significantly increase the luciferase activity in LoVo and RKO cells relative to counterpart cells (Figure 5h-i). A concordant phenomenon was observed when RNA interference was conducted in LoVo and RKO cells (Figure 5j-k).

### miR-433 modulates cell cycle progression and epithelial-mesenchymal transition

**Figure 6:**
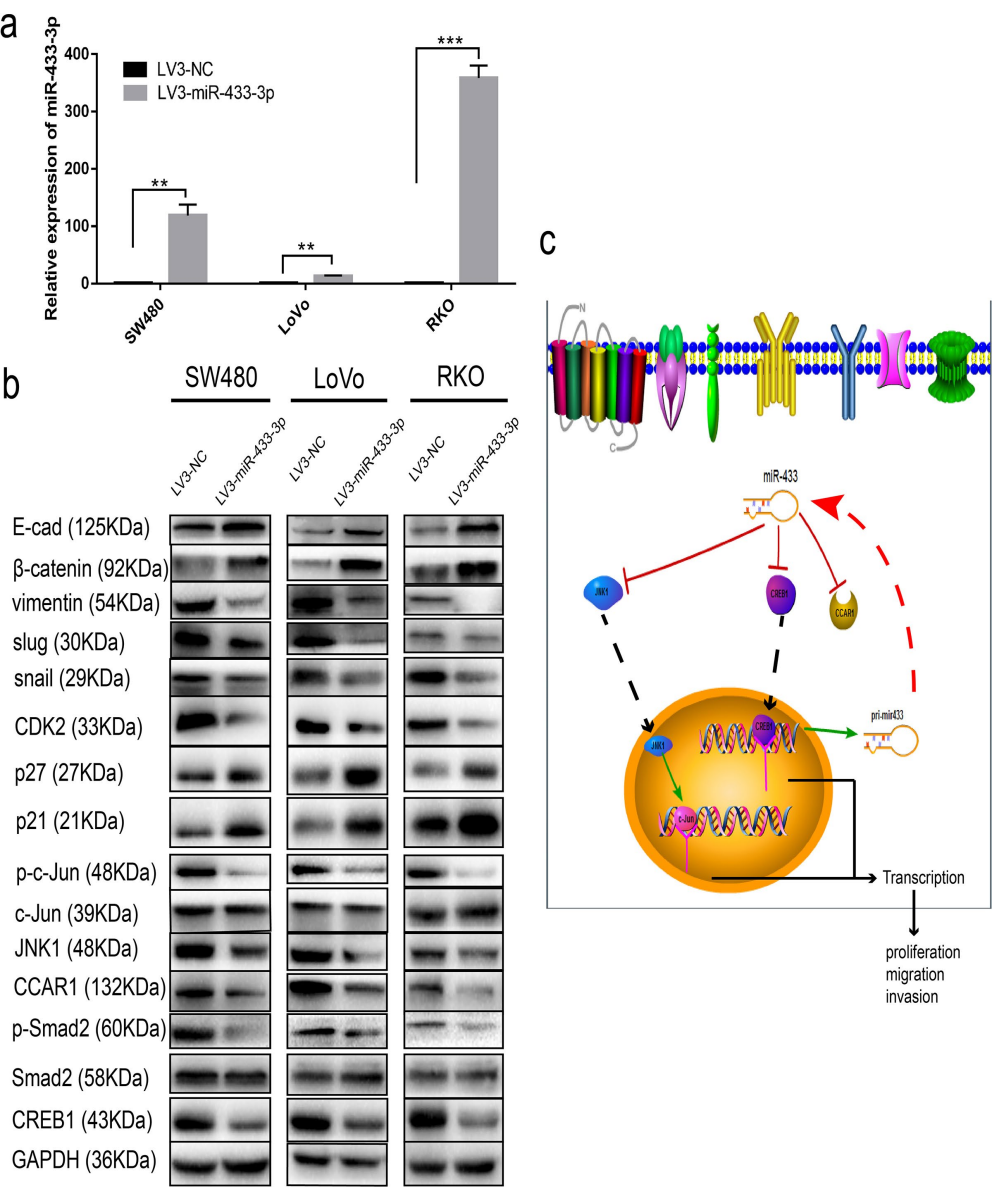
**miR-433 attenuated cell cycle progression and abolished epithelial-mesenchymal transition.** (**a**) CRC cells stably transduced with LV3-miR-433-3p displayed a notable upregulation of miR-433 compared with the control cells. (**b**) Western blotting showed downregulation of CREB1, CCAR1, and JNK1 and subsequent dephosphorylation of phospho-Smad2 and phospho-c-Jun, which in turn constrained cell cycle progression and EMT when cells were successfully infected with LV3-miR-433-3p. (**c**) Schematic diagram highlighting the mechanism of miR-433 in CRC. **, *p*<0.01; ***, *p*<0.001.

To determine the specific pathway underlying miR-433 involvement in CRC proliferation and metastasis, we detected some specific markers. As shown in Figure 6a, SW480, LoVo and RKO cells stably transduced with LV3-miR-433-3p presented much higher expression of miR-433-3p compared with their counterparts. Overexpression of miR-433-3p inhibited CREB1, CCAR1 and JNK1 expression in CRC cells, with an accompanying downregulation of phospho-Smad2, phospho-c-Jun, CDK2, vimentin, snail and slug and upregulation of p21, p27, E-cadherin and β-catenin (Figure 6b). Last, as demonstrated in the schematic in Figure 6c, CREB1 and miR-433 composed a feedback loop to regulate CRC proliferation and metastasis by targeting CCAR1 and JNK1.

## DISCUSSION

Although some studies have indicated the prognostic value of miR-433 among several types of malignancies, the mechanisms underlying its involvement in cancer metastasis and recurrence seem to be controversial. On the one hand, Gotanda and colleagues reported that miR-433 increased sensitivity to 5-FU in HeLa cells by regulating of thymidylate synthase [19]. However, Yu et al. and Weiner-Gorzel et al. respectively demonstrated that miR-433 could promote resistance to gemcitabine or paclitaxel in gallbladder and ovarian cancer cells [20,21]. On the other hand, Lin and colleagues observed that miR-433 suppressed hematopoietic cell differentiation in myeloproliferative neoplasms [22]. However, Tang et al. reported that miR-433 could promote osteoblast differentiation [23]. The heterogeneity of cancer could be one reason accounting for the disparity. In addition, given the fact that multiple targets co-exists for a single miRNA, it’s important to uncover and mine the driver targets involved in cancer progression. In this study, we conducted KEGG analyses in the miRWalk database and found that miR-433 was correlative with the MAPK and cAMP signaling pathways and cell adhesion molecules in colorectal cancer. In light of that JNK1 and CREB1 both are key mediator in the MAPK and cAMP signaling pathways, CCAR1 is viewed as a coactivator of β-catenin, we next explored the association between CREB1, CCAR1, JNK1 and miR-433.

Conventionally, CREB1 participates in the regulation of cell energy metabolism, cell proliferation, differentiation and cell survival as a transcription factor. Recent studies have shown that it may have a wider role. Corvaisier and colleagues revealed that the phosphorylation of CREB1 was engaged in the chemoresistance and colon cancer relapse [10]. Meanwhile, CREB1 was confirmed to crosstalk with other pathways. Rodón et al. observed that CREB1 could promote a malignant transforming growth factor (TGF) β2 autocrine loop in glioblastoma [24,25], which in turn induced epithelial–mesenchymal transition and endowed cancer cells with metastatic properties [26]. Our results manifested that miR-433 inactivated Smad2 via attenuation of its phosphorylation, thus abolishing EMT by targeting CREB1, which is accordant with the literature. In addition, CREB1 is involved in the regulation of K-RAS and mismatch repair (MMR) genes [27,28], which are vital molecular biomarkers related to targeted- and immune-therapy in CRC. Therefore, CREB1 is a potential treatment target [7]. In the study, we demonstrated that CREB1 is upregulated and negatively associated with miR-433 expression in CRC by real-time PCR. Patients with higher expression of CREB1 presented a worse OS through Kaplan-Meier analysis. Bioinformatics, western blot joint with luciferase reporter assay confirmed that CREB1 served as a direct target of miR-433. Simultaneously, CREB1 could transactivate miR-433 expression by ChIP and luciferase report assays. Taken together, aforesaid results unraveled our hypothesis that a reciprocal regulating relationship existed between miR-433 and CREB1.

Traditionally, CCAR1 (cell division cycle and apoptosis regulator 1) was viewed as a coactivator of β-catenin, p53 or nuclear receptors, dependent on disparate microenvironment, and responsible for regulating the expression of key proliferation- inducing genes [29]. Recently, CCAR1 showed a significant correlation with microvascular invasion, intrahepatic metastasis, higher T stage, and early recurrence in HCC, and could act as an independent predictor of shorter RFS (relapse-free survival) [30,31]. Moreover, depletion of CCAR1 lead to a significant reduction in the proliferation, migration and invasion phenotype in prostate, gastric and colorectal cancers [32–34]. Our study also demonstrated that CCAR1 is overexpressed in CRC compared with normal mucosa; is correlative to tumor depth, distant metastasis and pTNM stage; and could be an independent prognosticator.

JNK1 (c-Jun N-terminal protein kinase 1, MAPK8, JNK) is a canonical mediator in the SAPK/JNK signaling pathway and regulates many physiological processes, including inflammatory responses, morphogenesis, cell proliferation, differentiation, survival and death [35,36]. Recent studies have indicated that JNK1 can induce chemoresistance and enhance the viability and migration of cancer cells in gastrointestinal malignancies. Zhu and colleagues reported that the JNK1/c-Jun signaling pathway was involved in multidrug resistance in colon cancer cells [37]. Jemaà et al. demonstrated that JNK1 but not JNK2 is a downstream effector target in cancer cell migration and the effect of JNK inhibition in the metastatic potential of colon cancer cells [38]. Consequently, JNK1 is a promising therapeutic molecular target for an eventual cancer cure [39,40]. Our study denoted that JNK1 was upregulated in CRC; significantly associated with lymph node invasion, pTNM stage and CA19-9; and could serve as an independent predictor in prognosis. miR-433 targeted JNK1, and subsequently dephosphorylated phospho-c-Jun, thereby modulating CRC cells migration and invasion properties.

Meanwhile, some limitations existed in our work, an RNA-Seq approach may be useful for more precision and specific targets mining after alteration of miR-433 expression in CRC cell lines. The PDX (patient derived xenograft) mouse model combined with injection of miR-433 mimetic oligonucleotide (Agomir) will help to facilitate the process of its preclinical application.

## CONCLUSION

In sum, we report the presence of a reciprocal feedback loop between CREB1 and miR-433. miR-433 suppresses CRC proliferation, invasion and metastasis in vitro and in vivo through inhibition of cell cycle progression and EMT by targeting CREB1, CCAR1 and JNK1.

## MATERIALS AND METHODS

### Bioinformatics

The LinkedOmics (www.linkedomics.org) database was used to plot a survival curve according to the median of miR-433 expression. The miRWalk (http://mirwalk.umm.uni-heidelberg.de/) dataset was adopted to conduct KEGG pathway analysis. The Oncomine (www.oncomine.org) database was applied to explore the expression of CREB1, CCAR1 and JNK1 in CRC specimens. The TargetScan (www.targetscan.org) dataset was employed to predict the targets of miR-433 and its binding sequences. UCSC gene browser allied DBTSS (https://dbtss.hgc.jp), JASPAR, CONSITE (http://consite.genereg.net/) and LASAGNA-Search 2.0 (http://biogrid-lasagna.engr.uconn.edu/lasagna_search/) were utilized to discover the putative promotor region of miR-433 and potential transcription factor binding sites (TFBS).

### Patients and study materials

Thirty-five paired fresh colorectal cancer and adjacent normal mucosa tissues from patients who were diagnosed with adenocarcinoma of the colon or rectum in histology were collected in the Department of General Surgery, Shanghai Jiao Tong University Affiliated Sixth People’s Hospital with the informed consent of the patients. Another two hundred FFPE (formalin-fixed and paraffin-embedded) CRC patient sections between January 2010 and January 2012 were acquired from the Department of Pathology, Shanghai Jiao Tong University Affiliated Sixth People’s Hospital. Patients with incomplete follow-up were excluded. The histological type was determined as well, moderately or poorly differentiated. Tumor stage and pathological classification were defined according to the American Joint Committee on Cancer/Union for International Cancer Control 8th Edition. 164 patients (stage I-III) received radical colectomy or proctectomy and lymphadenectomy. Other 36 metastatic CRC patients received palliative chemotherapy or best supportive care. Postoperative adjuvant chemotherapy was given to 97 patients with pathological T4 tumors, T3 tumors with high-risk factors (poorly differentiated histology; lymphatic/vascular invasion; bowel obstruction; <12 lymph nodes examined; perineural invasion; localized perforation; indeterminate or positive margins) for relapse or lymph node metastasis. Among these 97 patients, 74 (stage III) were treated with FOLFOX (5-fluoro-2,4(1H, 3H)-pyrimidinedione, leucovorin, oxaliplatin), XELOX (xeloda, oxaliplatin) or FOLFIRI (5-fluoro-2,4(1H, 3H)-pyrimidinedione, leucovorin, irinotecan) regimens, and another 23 stage II patients with high-risk factors for recurrence were treated with xeloda, XELOX or FOLFOX regimens. The median follow-up time was 34 months (range: 1–88). A total of 130 patients died of CRC-related causes during the study period. Written informed consent was obtained from all patients in accordance with the local ethical guidelines. The whole procedure was approved by the Ethics Committee of Shanghai Jiao Tong University Affiliated Sixth People’s Hospital.

### Cell culture

The human CRC cell lines SW480, SW620, LoVo, HCT116, LS174-T, Caco-2, DLD-1, RKO and SW1116 were purchased from Cell Bank of Chinese Academy of Sciences (Shanghai, China) and cultured in Dulbecco’s modified Eagle’s medium (DMEM) (Gibco, Carlsbad, USA) supplemented with 10% fetal bovine serum (FBS) (Gibco, Carlsbad, USA) and 1% penicillin/streptomycin (KeyGen BioTECH, Nanking, China) in a humidified atmosphere of 5% CO2 at 37°C.

### Oligonucleotides transfection and lentivirus infection

miR-433 mimics, inhibitor, their respective negative controls, and the siRNA of CREB1 were purchased form GenePharma (Soochow, China) and successfully transfected into the corresponding cells according to the manufacturer’s instructions in the presence of Lipofectamine 2000 (Life Technologies, Carlsbad, USA). Similarly, the lentivirus packages of miR-433 and CREB1 overexpression vectors were also purchased from GenePharma, and successfully infected CRC cells by following the instructions.

### RNA isolation and quantitative real-time PCR

One milliliter of TRI Reagent^®^ (Sigma, St. Louis, MO, USA) was applied to dissociate the specimens and cells. Isopropanol (Sangon Biotech, Shanghai, China) was used to precipitate the total RNA. After measuring the concentration of RNA with a spectrophotometer (Tiangen, Peking, China), 1000 ng of total RNA was reverse transcribed to cDNA, and then, 1 µl cDNA was employed to conduct quantitative PCR (polymerase chain reaction) following the instructions of the manufacture (GeneCopoeia, Rockville, MD, USA). The specific primers of CREB1, CCAR1, JNK1, GAPDH, miR-433 and snRNA U6 were also purchased from GeneCopoeia. The 2^-△△CT^ method was adopted to analyze the relative expression.

### Western blotting

After 48-72 hours of relevant treatment, the cultured cells were washed twice with PBS (phosphate-buffered solution), and then, 50-100 µl RIPA lysis buffer (Sangon Biotech, Shanghai, China) supplemented with PMSF (phenylmethane sulfonyl fluoride), phosphatase and protease inhibitor (KeyGEN BIOTECH, Nanking, China) was used to elute the total protein. After measurement of protein concentration with a BCA (bicinchoninic acid) kit (Merck Millipore, Darmstadt, Germany), 50-100 µg of total protein was loaded and separated on a 10% SDS polyacrylamide gel, transferred to a PVDF (polyvinylidene difluoride) membranes, and incubated with anti-p21, anti-p27, anti-CDK2, anti-phospho-c-Jun or anti-phospho-Smad2 monoclonal rabbit antibody (1:1000, CST, Danvers, MA, USA); anti-E-cadherin, anti-β-catenin, anti-vimentin, anti-snail, anti-slug, anti-c-Jun, anti-Smad2, anti-CREB1, anti-JNK1 or anti-GAPDH rabbit polyclonal antibody (1:1000-1:5000, Proteintech, Chicago, IL, USA); or anti-CCAR1 rabbit polyclonal antibody (1:1000, SAB, Baltimore, MD, USA) overnight at 4°C. Protein bands were visualized using a chemiluminescence kit (Merck Millipore, Darmstadt, Germany).

### Immunochemistry

FFPE slides were heated at 60°C for two hours and then submerged in xylene for 10 mins (minutes) three times, ethyl alcohol twice for 5 mins, 95% ethanol, 85% ethanol, 75% ethanol for 5 mins, respectively. Then, antigen retrieval was performed with 10 nM citrate antigen retrieval solution (Sangon Biotech, Shanghai, China) in a microwave oven. After endogenous peroxidase inhibition and blocking procedures completed, the sections were incubated with anti-CREB1 rabbit polyclonal antibody (1:400, Proteintech, Chicago, IL, USA), anti-CCAR1 rabbit polyclonal antibody (1:400, SAB, Baltimore, MD, USA) and anti-JNK1 rabbit polyclonal antibody (1:300, Proteintech, Chicago, IL, USA). The other procedures, H&E (hematoxylin & eosin) staining and immune scoring of the slides were conducted as described in our previous work [41].

### Dual luciferase reporter assay

To determine the binding between miR-433 and its targets, a certain number of HEK293T cells were seeded in a 24-well plate in advance, and then the cells were co-transfected with 20 pmol of miR-433 mimics or negative control with wild-type (wt) or mutant (mut) 3’UTR vectors using Lipofectamine 2000. After 48 hours, the cells were washed with PBS twice and lysed with passive lysis buffer (Promega, Madison, WI, USA). For the binding between CREB1 and the mir433 promoter, LV5-NC- and LV5-CREB1-infected cells were similarly counted and seeded in a 24-well plate prior to the experiment, and concurrently, the relevant cells were pre-transfected with CREB1 siRNA and the negative control 24 hours prior to the experiment, and then, the CRC cells were transfected with wt and mut mir433 promoter dual luciferase plasmids accordingly in the presence of Lipofectamine 2000. The subsequent procedures were performed following the instructions of the manufacturer (Promega, Madison, WI, USA). At last, the luciferase activity was collected with a microplate reader (BioTeke, Peking, China).

### Chromatin immunoprecipitation (ChIP) assay

For the ChIP assay, CRC cells were infected with the CREB1 overexpression lentivirus LV5-CREB1. Anti-CREB1 polyclonal antibody (Abcam, Cambridge, UK) and a Chromatin Immunoprecipitation (ChIP) Assay Kit (Merck Millipore, Darmstadt, Germany) were adopted in this assay. Other procedures were performed as described previously [42].

### MTT assay

After relevant treatments were conducted, the CRC cells were counted and adjusted to a concentration of 1 x 10^6^/ml, and 1 x 10^3^ cells per well were seeded into a 96-well plate. Then, 50 µl of 1 x MTT (methyl thiazolyl tetrazolium) reagent (KeyGen BioTECH, Nanking, China) was added into the medium accordingly. The OD (optical density) value was obtained with a microplate reader.

### Transwell assay

Similarly, after treatment, 1-2 x 10^5^ CRC cells were seeded in the upper chamber of 8-µm polycarbonate membrane filters (Corning, NY, USA) with or without Matrigel (Corning, NY, USA), and 600 µl of medium containing 10% FBS as a chemokine was placed in the lower chamber. After 24-48 hours, the cells in the lower chamber were fixed in 4% paraformaldehyde and stained with 0.5% crystal violet. Then, five random fields were photographed.

### Colony formation assay

CRC cells from different treatment groups were seeded in 6-well plates (1 x 10^3^ cells/well) and then cultured for two weeks. After, the cells were fixed in pre-cooled formaldehyde and stained with 0.5% crystal violet. The colony numbers were counted accordingly.

### Subcutaneous tumorigenicity and liver metastasis mouse model

Approximately 5 x 10^6^ CRC cells were inoculated into the right flank of female BALB/c nu-nu mice. The mice were observed every week. After optical tumor appeared, a vernier caliper was applied to measure the length and width each week. Eight weeks later, the mice were sacrificed, and the tumors were collected and weighed. For the CRC liver metastasis model, a 1-cm single incision was made in the upper left lateral abdomen of the mice to expose the spleen in the intraperitoneal administration of 10% chloral hydrate. The other procedures were followed according to a classic method reported previously [43]. All animal experimental procedures were approved by the Ethics Committee of Shanghai Jiao Tong University Affiliated Sixth People’s Hospital and the mice were cared in accordance with the institution guidelines.

### Statistical analyses

All data were analyzed through SPSS13.0 or GraphPad Prism 7.0 software using Student’s *t* test, χ^2^ test or one-way analysis of variance (ANOVA) as appropriate and are presented as the mean ± standard deviation (SD). Spearman correlation was adopted to evaluate the relationship between miR-433 and its targets. The Cox proportional hazard regression model and Kaplan-Meier analyses were applied to identify the independent prognostic variables and to plot survival curves using a log-rank test. *p*<0.05 was considered statistically significant.

## SUPPLEMENTARY MATERIAL

Supplementary Figure S1

Supplementary Figure S2

Supplementary Figure S3
