# Supplementary material for: A CREB1/miR-433 reciprocal feedback loop modulates proliferation and metastasis in colorectal cancer
Source: Aging (Albany NY). 2018 Dec 6;10(12):3774–93. doi: 10.18632/aging.101671 (PMC6326693; doi:10.18632/aging.101671)
Supplement: Supplementary Figure S1 [file aging-10-101671-s001.pdf]

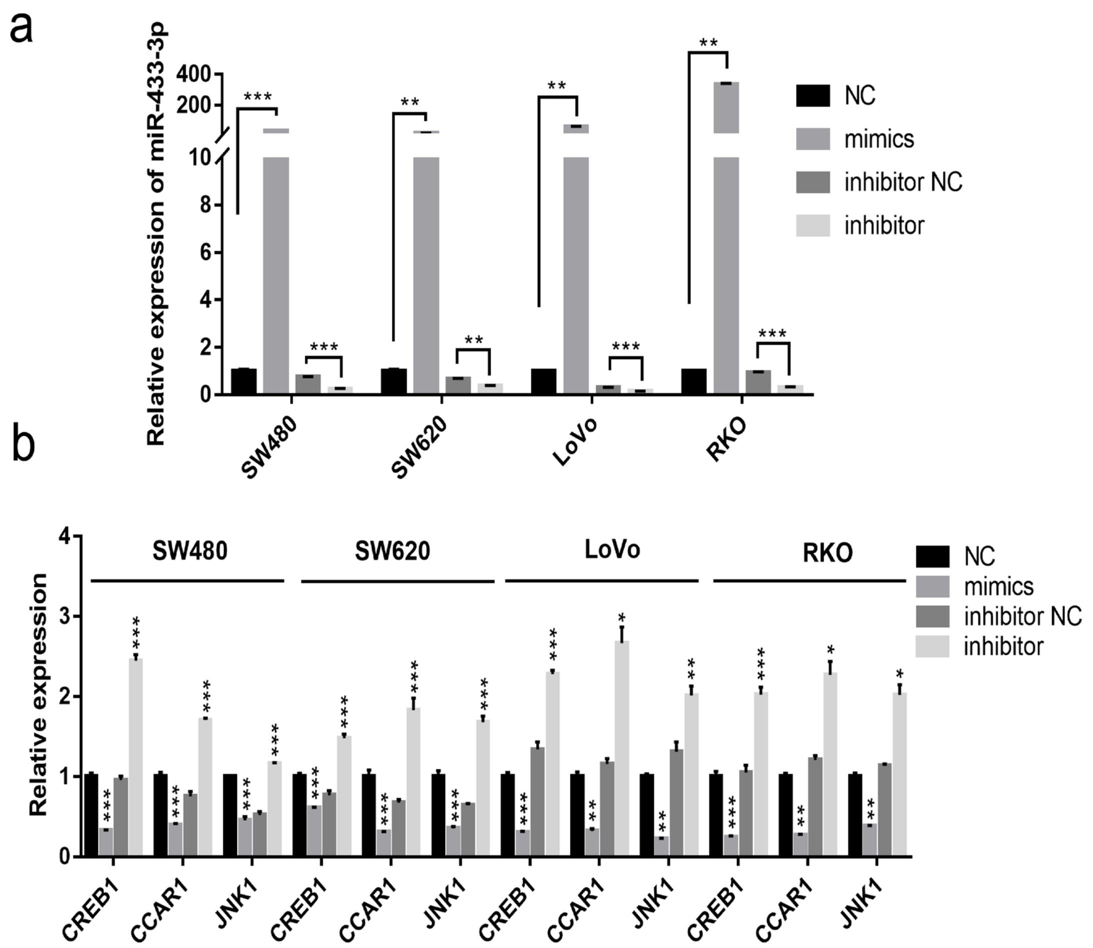

Supplementary Figure S1. Successful transient transfection of CRC cells with miR-433. (**a**) The relative expression of miR-433-3p after SW480, SW620, LoVo and RKO cells were transfected with an miR-433-3p mimics or inhibitor. (**b**) The expression changes in CREB1, CCAR1 and JNK1 after cell transfection. NC, negative control; mimics, miR-433 mimics; inhibitor NC, negative control for inhibitor; inhibitor, inhibitor of miR-433. *, p<0.05; **, p<0.01; ***, p<0.001.
