# Supplementary material for: A CREB1/miR-433 reciprocal feedback loop modulates proliferation and metastasis in colorectal cancer
Source: Aging (Albany NY). 2018 Dec 6;10(12):3774–93. doi: 10.18632/aging.101671 (PMC6326693; doi:10.18632/aging.101671)
Supplement: Supplementary Figure S2 [file aging-10-101671-s002.pdf]

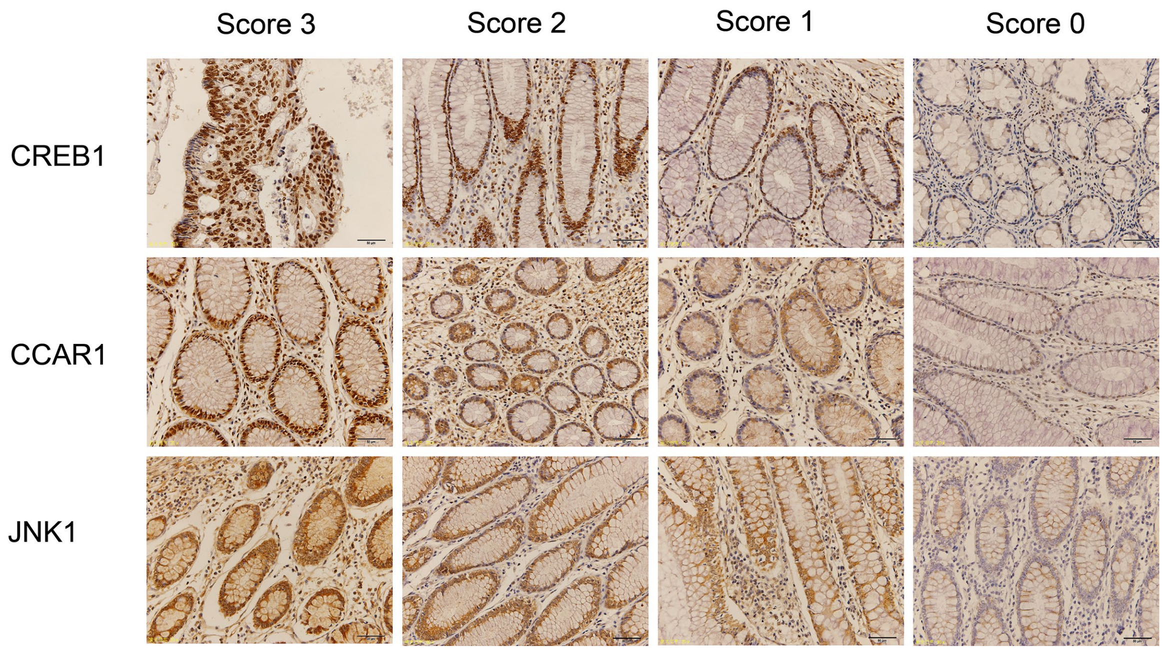

Supplementary Figure S2. Representative high-power field IHC images of CREB1, CCAR1 and JNK1 staining are presented according to the stratified intensity.
