# Supplementary material for: A CREB1/miR-433 reciprocal feedback loop modulates proliferation and metastasis in colorectal cancer
Source: Aging (Albany NY). 2018 Dec 6;10(12):3774–93. doi: 10.18632/aging.101671 (PMC6326693; doi:10.18632/aging.101671)
Supplement: Supplementary Figure S3 [file aging-10-101671-s003.pdf]

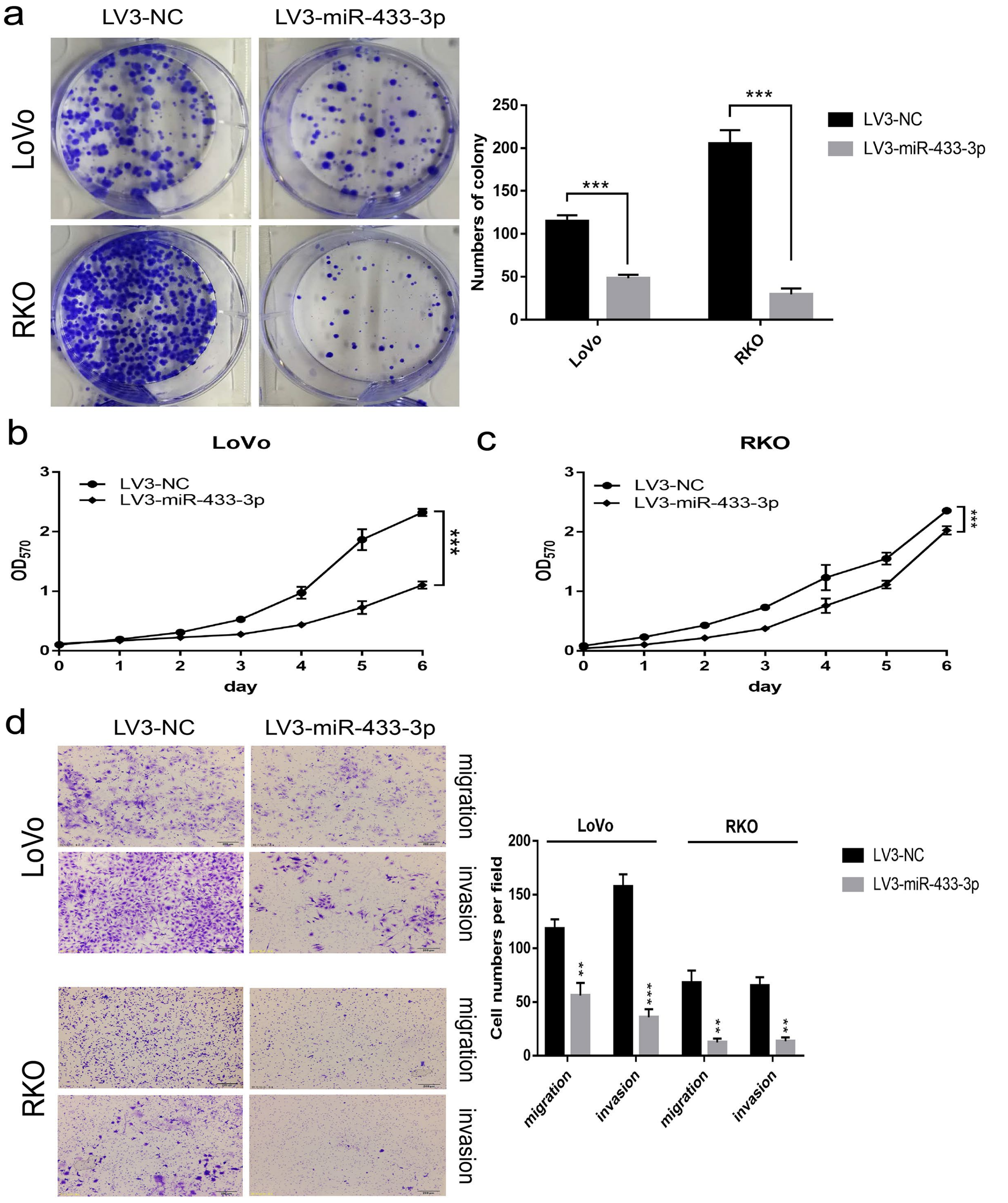


**Supplementary Figure S3. LV3-miR-433-3p infection suppressed proliferation, migration and invasion in LoVo and RKO cells.** (**a**) LV3-miR-433-3p infection alleviated the colony formation activity of CRC cells. LV3-miR-433-3p reduced the cell viability in LoVo (**b**) and RKO (**c**) cells. (**d**) LV3-miR-433-3p restrained the migration and invasion of CRC cells. **, *p*<0.01; ***, *p*<0.001.
